# Anxiety and depressive symptoms as predictors of substance use initiation among adolescents living on and near a Tribal reservation in the Great Plains region of the U.S.

**DOI:** 10.3389/frcha.2024.1390793

**Published:** 2024-11-18

**Authors:** Caroline M. Barry, Ashna Jagtiani, Melvin D. Livingston, Sierra Talavera-Brown, Hannah LaBounty, Eugena Atkinson, Juli R. Skinner, Kelli A. Komro

**Affiliations:** ^1^ Rollins School of Public Health, Behavioral, Social, and Health Education Sciences, Emory University, Atlanta, GA, United States; ^2^ Cherokee Nation Behavioral Health, Cherokee Nation Health Services, Tahlequah, OK, United States

**Keywords:** substance use initiation, anxiety symptoms, depressive symptoms, internalizing, American Indian, adolescence, longitudinal

## Abstract

**Introduction:**

This study examines the impacts of anxiety and depressive symptoms on subsequent initiation of alcohol use, cannabis use, and prescription opioid misuse among diverse adolescents attending high schools on or near a Tribal reservation in a rural Great Plains region of the U.S.

**Methods:**

In collaboration with Emory University and a Great Plains Tribal nation's behavioral health organization, a community randomized trial of 20 high schools was conducted to prevent substance misuse. Surveys administered at four time points (fall and spring of 10th and 11th grade) included the GAD-7, PHQ-8, and items assessing lifetime alcohol use, cannabis use, prescription opioid misuse, and covariates (age, gender, race, and food insecurity). The analytic sample included students with data at two or more time points (*n* = 455) from control schools (*k* = 10). Approximately half of the sample identified as American Indian only or American Indian/White only, and 36%–39% as White only.

**Results:**

Adjusted generalized estimating equations showed that every 5-point increase in anxiety symptoms was associated with 1.28 and 1.29 times the odds of initiating alcohol and cannabis use respectively the following semester. Similarly, every 5-point increase in depressive symptoms was associated with 1.25, 1.34, and 1.38 times the odds of initiating alcohol use, cannabis use, and prescription opioid misuse respectively the following semester.

**Discussion:**

Results show a consistent 25%–38% increased odds of certain types of substance use initiation following increases in anxiety and depressive symptoms among adolescents. Findings underscore the need for targeted prevention and intervention to address mental health issues among a historically marginalized population. Addressing mental health concerns earlier may mitigate later substance use risks and sequelae for rural and American Indian youth.

## 1 Introduction

There is a crisis of mental health and substance use problems among adolescents and young adults, with an alarming lack of services to prevent escalation and further mental, social, and physical sequelae (1–3). The prevalence of clinically elevated anxiety and depressive symptoms among youth globally were 20.5% and 25.2% respectively during the COVID-19 pandemic years (4). Similarly, rates of substance use are concerning and often co-occur with mental health problems (5). According to results of the 2022 Monitoring the Future Survey, among 10th graders in the U.S., the most commonly used substances are alcohol (31.3%), nicotine vapes (20.5%), and cannabis (19.5%), followed by other drugs (6). Further, the prevalence of unmet mental and behavioral health care needs among adolescents in the U.S. is estimated at 60% (1).

Patterns of risk among American Indian (AI) adolescents are of particular concern, as are barriers to preventive mental and behavioral health services for AI and other adolescents living in rural communities (7–10). American Indian/Alaska Native (AI/AN) populations experience some of the highest suicide rates in the U.S., with the incidence of suicide nearly twice as high as the national average, which is exacerbated by co-occurring substance use and limited access to mental health care (8–11). This crisis is compounded by the alarming rise in drug overdose deaths among AI/AN adolescents and young adults, largely attributable to opioids and synthetic substances like fentanyl, with overdose mortality rates increasing more rapidly for this group than for other racial/ethnic groups over the past two decades (7). Structural barriers are common obstacles experienced by rural adolescents, including provider shortages, lack of transportation, inconvenient hours, and unaffordable costs (2). Internalized barriers further impede the likelihood of rural AI adolescents accessing treatment services for mental health and substance use problems, including self-reliance, concerns of compromised privacy and judgment from others, lack of trust in providers, fear of provider discrimination, and unfavorable perceptions of service quality and relevance (8, 9). Despite these pressing concerns, it is important to acknowledge the strength, resilience, and cultural traditions that many youth, specifically those from AI communities, draw upon to navigate these challenges (10, 12). AI communities possess deep cultural strengths, including strong community bonds and traditional practices that serve as protective factors for mental health and substance use prevention. These strengths are often underleveraged in mainstream health interventions, and community-specific research is needed to inform culturally relevant prevention strategies (10, 12).

Evidence shows that on average, substance use initiation occurs between ages 15–17 years, with moderate correlations between substances and differences in use trajectories based on age and type of substance at initiation (13, 14). Age at first use of alcohol and other drugs is predictive of later use problems as well as progression to disordered use and dependence (15–17). Compared to initiation during adulthood, initiation of substance use during adolescence is associated with a greater likelihood of other adverse outcomes due to neurodevelopmental disruption and dysregulation of mechanisms in the maturing brain (18). Early substance use onset predicts a range of deleterious consequences, including mental, social, and physical problems, increased risk behaviors, disordered substance use, and worse academic performance (15, 19–22). Cannabis use during adolescence has been linked to a higher risk of subsequent mood disorders, psychosis, and drug addictions into adulthood (18). In rural communities and Tribal reservations, AI adolescents have engaged in significantly earlier initiation of alcohol intoxication, cannabis use, and inhalant use compared to white counterparts (23). A study of initiation patterns among AI adolescents of a Northern Plains tribe revealed that cannabis was the most common first substance of use, before alcohol, which may reflect cultural factors and a changing landscape of legalization and availability of cannabis (24). Research on AI adolescents has shown there may be a significantly increased risk of escalation to using illicit drugs like cocaine within five years following initiation of alcohol use, cannabis use, or inhalant use (25). By delaying substance use initiation, prevention strategies may effectively mitigate progression to substance use problems (26).

Given the prevalent co-occurrence of mental health and substance use problems during adolescence, identifying causal pathways has become an important focus to inform intervention approaches (27). Theoretically, two main pathways have been proposed: the substance-induced pathway, whereby substance use elicits mental health problems, and the symptom-induced pathway, through which mental health problems lead to substance use (28–30). The evidence, although mixed, suggests a degree of bidirectionality between a single mental health issue or cluster of problems (e.g., internalizing vs. externalizing symptoms) and ongoing substance use (29–31). Externalizing symptoms, encompassing behaviors like aggression, delinquency, or deviance, are consistently highlighted as reliable predictors of substance use and related disorders (21, 32–34). However, recent attention has shifted towards an internalizing pathway to substance use, where individuals turn to substances to alleviate negative emotions like anxiety and depression (35–37). Despite being less convincingly supported compared to externalizing symptoms, theoretical assumptions of a causal link between internalizing symptoms and substance use among adolescents persist in the literature (29, 38).

Anxiety symptoms may be linked to substance use problems, but evidence is inconsistent (31). A 2018 meta-analysis of 36 studies of adolescents and young adults in North America found that clinical anxiety was significantly associated with the use of alcohol, cannabis, and tobacco products at a cross-section in time but not longitudinally (5). Including subclinical symptoms and specific subtypes of anxiety, another recent systematic review of 51 prospective cohort studies showed some evidence of a longitudinal association between anxiety in youth and later alcohol use disorders, but meta-analysis showed no significant association on alcohol use disorders or outcomes of alcohol consumption, and initiation outcomes were not examined (35). Fewer studies have examined anxiety and illicit drug use, but tested associations have been null (31). Of studies examining initiation, significant predictors of alcohol use initiation include early anxiety symptoms in childhood (32), situationally induced anxiety, and social anxiety (33). However, anxiety has shown protective effects against substance use in an equivalent number of studies (31). For example, social anxiety was found to be protective against the initiation of cannabis use among high school students (34). Anxiety symptoms have been associated with a lower risk of initiating alcohol use among male adolescents (36), and panic and separation anxiety symptoms have been associated with a lower risk of alcohol use initiation among a sample of young adolescents (37). These nuanced findings underscore the complexity of the relationship between anxiety symptoms and substance use initiation, emphasizing the need for targeted research to elucidate the specific conditions under which anxiety may act as a risk or protective factor.

Compared to anxiety, depressive symptoms are more consistently associated with substance use and related problems (31). The 2018 meta-analysis mentioned previously revealed significant cross-sectional associations between clinical depression and the use of alcohol, cannabis, and tobacco products, as well as a longitudinal association between depression and tobacco use (5). Depressive symptoms have also been found to predict tobacco smoking initiation in adolescence and are considered a more reliable predictor of alcohol use than anxiety (31, 38–40). Growing evidence on opioid outcomes shows that depression, mental health problems (including affective, conduct, and behavioral disorders), and mental health-related hospitalization are all significantly associated with opioid misuse in adolescence (41). However, there is no longitudinal research examining anxiety and depressive symptoms as predictors of initiation of alcohol use, cannabis use, and prescription opioid misuse among rural AI and other youth despite a disproportionate health burden and history of marginalization. The absence of longitudinal research in this specific context highlights a critical gap that warrants further exploration.

To begin to address this gap, data for the current study came from the control group of a cluster-randomized prevention trial on and near a Tribal reservation in the Great Plains region of the U.S. (42). Our objective was to examine the longitudinal associations of mental health symptoms on the initiation of substance use among a sizable population of AI and rural adolescents. Specifically, we examined the distinct effects of anxiety symptoms and depressive symptoms on subsequent initiation of alcohol use, cannabis use, and prescription opioid misuse using generalized estimating equations. We also examined potential moderation of these relationships by gender and race given previously documented group differences (43, 44).

## 2 Method

### 2.1 Study design and sample

A prevention trial to reduce substance use among high school students was conducted collaboratively between Emory University and a Tribal behavioral health organization, a leader in prevention and clinical services in the Great Plains. As described in the trial protocol (42), high schools were eligible to participate if they were located in counties overlapping with the Tribal nation's reservation, had town populations of 3,000 or fewer, and class sizes ranging from 30 to 100 students. Schools were excluded if they were situated in metropolitan or micropolitan core areas, as defined by the U.S. Census Bureau's Rural-Urban Commuting Area codes, or if they had an existing community drug prevention coalition. Administrators from 20 high schools meeting these criteria agreed to enroll their schools and undergo randomization to receive intervention strategies for prevention and support during the trial period (intervention group) or after the trial (delayed-intervention control group). Students in both study groups completed identical surveys on tablets during school hours at the same four time points: T1 (Fall 2021, 10th Grade), T2 (Spring 2022, 10th Grade), T3 (Fall 2022, 11th Grade), and T4 (Spring 2023, 11th grade). The survey included measures of mental health symptoms, alcohol use, cannabis use, prescription opioid misuse, and sociodemographic characteristics. Additional details about the trial design and other measures have been described in the trial protocol and measurement papers (42, 45). Study materials, methods, and this manuscript have been approved by the Tribal nation's IRB. The Tribal nation's IRB is the acting IRB for this study, Emory University IRB has signed a reliance agreement, and the neighboring Tribal nation has provided an agreement letter. The sample for this analysis included students from the control group schools (*k* = 10) who completed the survey at two or more time points (*n* = 455).

### 2.2 Measures

#### 2.2.1 Anxiety symptoms

Anxiety symptoms were measured with the Generalized Anxiety Disorder 7-item scale (GAD-7), which has been validated for use among the general population, adolescents, and our study population (45–47). Responses on the GAD-7 were indicated on a 4-point scale where 0 = not at all, 1 = several days, 2 = more than half the days, and 3 = nearly every day with a total score range from 0 to 21. Scores 5, 10, and 15 are the cutpoints for mild, moderate, and severe levels of anxiety, respectively (47).

#### 2.2.2 Depressive symptoms

Depressive symptoms were measured with the 8-item Patient Health Questionnaire (PHQ-8), which has been validated for use in the general population and in our study population (45, 48). Responses on the PHQ-8 were indicated on a 4-point scale where 0 = not at all, 1 = several days, 2 = more than half the days, and 3 = nearly every day with a total score range from 0 to 24. Scores 5, 10, 15, and 20 are the respective cutpoints for mild, moderate, moderately severe, and severe depressive symptoms (48).

#### 2.2.3 Substance use

Lifetime use of alcohol and marijuana (referred to as cannabis in the remainder of this paper, though measured using the term “marijuana” in the survey) and lifetime prescription opioid misuse (operationalized as “without a doctor's prescription or differently than how a doctor or medical provider told you to use it”) were assessed using three separate items with responses indicated on a 7-point scale where 0 = 0 times, 1 = 1–2 times, 3 = 3–9 times, 4 = 10–19 times, 5 = 20–39 times, 6 = 40–99 times, and 7 = 100 or more times. Binary (0 or 1) lifetime substance use variables were constructed based on these responses to capture the initiation of each substance.

#### 2.2.4 Sociodemographic covariates

Age, gender, race, and food insecurity were collected at all time points (42). For gender, response options included female, male, or decline to answer. For race, students were asked to select all that apply, resulting in four categories: American Indian (AI) only, American Indian and White only (AI/White), White only, and Other. Food insecurity, a proxy for socioeconomic status, was measured by asking students, “During the past 30 days, did you worry that food at home would run out before your family got money to buy more?” with three response options: 1 = a lot, 2 = sometimes, and 3 = never.

### 2.3 Sample construction

To capture initiation for each type of substance use, we followed these steps. First, we created a subset of students with no lifetime use at T1 (*n* = 227 students who had not used alcohol, *n* = 329 students who had not used cannabis, and *n* = 439 students who had not misused prescription opioids) and merged their GAD-7 and PHQ-8 scores at T1 with their lifetime substance use at T2. For those who had not initiated substance use at T2, T3 substance use was merged with T2 GAD-7 and PHQ-8 scores. An equivalent process was used to combine T4 substance use and T3 GAD-7 and PHQ-8 scores.

Overall, the sample construction included four waves of data collection from control schools: Wave 1 (*n* = 454), Wave 2 (*n* = 466), Wave 3 (*n* = 451), and Wave 4 (*n* = 410). The analysis required at least two time points to predict initiation of substance use, using GAD-7 and PHQ-8 scores from the prior wave to predict lifetime substance use at the subsequent wave. In cases where predictor data were missing at an earlier wave, data from the next available wave were used to establish each participant's baseline. These were then concatenated to create a single dataset for analysis.

### 2.4 Data analyses

All analyses were performed in RStudio version 4.2.0 (49). Means (SD) for all continuous variables and frequencies (%) for all categorical variables were calculated. To estimate the associations between mental health symptoms and substance use initiation, we estimated a series of logistic regressions. For each substance, we fit two separate multivariable models. Model 1 included the exposure (anxiety or depressive symptoms) and time point. The study time point was included to account for our pooling of multiple cross-sections over time. Model 2 included the exposure (anxiety or depressive symptoms), time point, baseline age, gender, race, and food insecurity. Additional models tested for moderation by gender and race. However, no statistically significant interactions were observed. The interactions were thus removed from the models. Log odds and robust standard errors were used to calculate odds ratios and 95% CIs for all models. For interpretation, anxiety, and depressive symptoms scores were rescaled such that odds ratios represent a 5-point increase on GAD-7 and PHQ-8 (equivalent to a one-category increase based on clinical cutpoints). Because students who have yet to initiate may be measured multiple times across study waves, we estimated all models as generalized estimating equations (GEE) with an exchangeable correlation structure clustered on the student ID. Complete case analysis was used to handle missing data. As a sensitivity analysis, we ran mixed-effects models including participants with missing data, and results did not differ substantially from those of the GEEs.

As an additional check, we compared participants in the analytic sample (*n* = 455) to those omitted (*n* = 108) based on our sample construction criteria, i.e., those with only one data time point or substance use at T1. We tested for significant differences on age, gender, race, food insecurity, and anxiety and depressive symptoms.

## 3 Results

Table 1 presents descriptive statistics for the non-users of each substance at baseline. The total numbers of students reporting no use of alcohol, no use of cannabis, and no misuse of prescription opioids at baseline were 227 (49.9%), 329 (72.3%), and 439 (96.5%), respectively, of 455 total students in the analytic sample.

**Table 1:** Baseline sample characteristics.

Characteristics	Alcohol non-initiators *n* = 227	Cannabis non-initiators *n* = 329	Prescription opioid non-initiators *n* = 439
Age	15.62 (0.79)	15.63 (0.72)	15.64 (0.69)
Gender			
Female	106 (46.7)	156 (47.4)	210 (47.8)
Male	109 (48.0)	159 (48.3)	215 (49.0)
Decline to answer	12 (5.3)	14 (4.3)	14 (3.2)
Race			
American Indian only	47 (20.7)	72 (21.9)	96 (21.9)
American Indian and White only	63 (27.8)	91 (27.7)	128 (29.2)
White only	84 (37.0)	126 (38.3)	157 (35.8)
Other	33 (14.5)	40 (12.2)	58 (13.2)
Food insecurity			
A lot	8 (3.5)	11 (3.3)	11 (2.5)
Sometimes	31 (13.7)	42 (12.8)	60 (13.7)
Never	188 (82.8)	276 (83.9)	368 (83.8)
Anxiety symptoms (GAD-7)	5.88 (5.64)	6.25 (5.69)	6.54 (5.69)
Depressive symptoms (PHQ-8)	6.50 (5.86)	6.75 (5.82)	7.35 (5.87)

Mean (SD) shown for continuous variables; frequency (%) shown for categorical variables. GAD-7, generalized anxiety disorder 7-item scale; PHQ-8, 8-item patient health questionnaire. Food insecurity was measured with the item, “During the past 30 days, did you worry that food at home would run out before your family got money to buy more?”

When controlling for covariates, every 5-point increase in anxiety symptoms was significantly associated with 1.28 (95% CI: 1.03, 1.59) times the odds of alcohol use initiation, while every 5-point increase in depressive symptoms was significantly associated with 1.25 (95% CI: 1.03, 1.52) times the odds of alcohol use initiation (Table 2).

**Table 2:** Mental health symptoms predicting odds of substance use initiation.

	Alcohol use initiation OR (95% CI)		Cannabis use initiation OR (95% CI)		Prescription opioid misuse initiation OR (95% CI)	
	Model 1	Model 2	Model 1	Model 2	Model 1	Model 2
Anxiety symptoms (GAD-7)	1.20 (0.99, 1.45)	**1.28** (**1.03, 1.59)**	**1.24** (**1.03, 1.50)**	**1.29** (**1.04, 1.59)**	1.11 (0.87, 1.42)	1.14 (0.88, 1.47)
Depressive symptoms (PHQ-8)	1.18 (0.98, 1.40)	**1.25** (**1.03, 1.52)**	**1.27** (**1.06, 1.52)**	**1.34** (**1.09, 1.64)**	**1.29** (**1.03, 1.62)**	**1.38** (**1.08, 1.75)**

Model 1, anxiety or depressive symptoms; Model 2, anxiety or depressive symptoms, age, gender, race, food insecurity, and time point; GAD-7, generalized anxiety disorder 7-item scale; PHQ-8, 8-item patient health questionnaire. GAD-7 and PHQ-8 scores were rescaled to represent a 5-point increase in their scales.

Bold indicates *p* < 0.05.

When controlling for covariates, every 5-point increase in anxiety symptoms was significantly associated with 1.29 (95% CI: 1.04, 1.59) times the odds of cannabis use initiation, while every 5-point increase in depressive symptoms was significantly associated with 1.34 (95% CI: 1.09, 1.64) times the odds of cannabis use initiation (Table 2).

When controlling for covariates, every 5-point increase in depressive symptoms was significantly associated with 1.38 (95% CI: 1.08, 1.75) times the odds of prescription opioid misuse initiation. Anxiety symptoms were not statistically significantly associated with opioid initiation (Table 2).

While no interactions by race and gender were observed, *post hoc* tests of the mean differences in anxiety and depressive symptoms scores by gender and race were performed. No significant differences were found on mental health scores by race, indicating that the burden of anxiety or depressive symptoms was relatively consistent across racial/ethnic groups in our sample. However, significant differences were found between genders (*p* < .0001 for both GAD-7 and PHQ-8). On the GAD-7, the mean (SD) for females, males, and those who declined to answer respectively were 8.53 (5.80), 4.23 (4.29), and 13.24 (6.37). On the PHQ-8, the mean (SD) for females, males, and those who declined to answer respectively were 9.00 (6.01), 5.12 (4.50), and 15.82 (5.74).

Additionally, results from tests comparing the analytic sample to those omitted showed significant differences: the omitted participants were older (mean age: 16.29 vs. 15.63, *p* < 0.001), reported more food insecurity (“a lot”: 11.2% vs. 2.9%, *p* < 0.001), and had higher GAD-7 (8.02 vs. 6.61, *p* < 0.05) and PHQ-8 scores (9.59 vs. 7.36, *p* < 0.001). No significant differences were found for gender or race.

## 4 Discussion

This is the first study to examine the longitudinal associations of anxiety and depressive symptoms on initiation of alcohol use, cannabis use, and prescription opioid misuse among AI and other adolescents living in rural areas. Results show a consistent 25%–38% increased odds of substance use initiation for every 5-point increase in anxiety or depressive symptoms across substances (in five of six adjusted models). Findings demonstrate compelling, consistent evidence in support of a symptom-induced pathway of substance use, suggesting that adolescents may turn to substances like alcohol or drugs as a maladaptive strategy for managing emotional distress. This process aligns with self-medication theory, which posits that individuals use substances to alleviate the psychological discomfort associated with internalizing symptoms or negative affect (28, 31, 50, 51). Lack of moderation by race and gender suggest relevance of significant associations across groups within our sample. Notably, adolescents identified as female or who declined to disclose their gender had higher reported anxiety and depressive symptoms, which may put them at increased risk of substance use initiation.

As presented earlier, research on the symptom-induced pathway shows mixed effects of anxiety and more consistent effects of depression. Most studies have assessed outcomes of ongoing substance use rather than initiation, and the majority of evidence comes from tobacco and alcohol research among non-Native populations. Critically, however, cannabis and prescription opioids are of increasing relevance given the rapidly changing legal landscape affecting availability and social norms. Since Oklahoma legalized medical cannabis in 2018, the state has experienced a proliferation of dispensaries. Many dispensaries market in a way that could modify adolescents' risk environments, including advertisements for product varieties (e.g., edibles, vape pens) and price promotions (52). However, recent research suggests that changes in cannabis legality may not significantly increase adolescent use, contrary to some concerns. Studies have shown that adolescent cannabis use did not rise substantially following legalization in Washington state and other regions up through 2020 (53–55). Nevertheless, increased marketing exposure may lead to increased cannabis use among adolescents, reduced harm expectancies, and increased perceptions of restorative or health-promoting properties that align with Native plant-based healing approaches (56, 57). Additionally, Tribal Nations and the state of Oklahoma recently reached multi-million-dollar settlements in lawsuits against pharmaceutical companies due to allegations that the companies contributed to the over-distribution of opioids throughout communities (58). Together, these contextual factors may partially explain a normative preference for self-medication with cannabis over prescription opioids.

The null finding concerning anxiety symptoms and prescription opioid misuse, while diverging from other results, is not entirely unexpected given the limited understanding of the anxiety-illicit drugs association. The lower prevalence of individuals initiating prescription opioid misuse (3.5% at baseline in the analytic sample) may contribute to challenges in detecting effects on this outcome.

This study has several strengths. The longitudinal analysis allowed us to establish temporality between mental health as the exposure and substance use initiation as the outcome. Additionally, the restriction of the sample to baseline non-users ensured we captured initiation among the analytic sample at subsequent time points. Baseline prevalences of substance use suggest that assessment before 10th grade may have helped catch earlier substance use initiation and the predictive power of mental health for those early initiators not represented in our analytic sample.

Despite these strengths, our study is not without limitations. First, data were susceptible to potential cohort effects during years of the COVID-19 pandemic and recent proliferation in cannabis dispensaries throughout the region (52), both of which could limit the generalizability of findings beyond our study population. Additionally, while the primary outcome measures have been quantitatively validated in this sample, the PHQ-8 and GAD-7 were not originally developed or conceptualized with AI contexts in mind. As such, the precision with which we captured culturally relevant expressions of mental health may be affected. However, there were minimal differences between adolescents who identified solely as AI, AI mixed race, and White only in the psychometric study of our measures in this population, which supports the reliability of interpreting results across these demographic groups (45). Further, our comparison of the analytic sample to those omitted showed that omitted participants were significantly older, more food insecure, and had higher levels of anxiety and depression. This suggests that early substance use initiators (before or during the fall of 10th grade) and those with incomplete data may have had more severe risk profiles on average. As a result, our findings may be more reflective of lower-risk, more stable youth, which could limit the generalizability of the results to higher-risk, harder-to-reach populations with greater food insecurity or more severe mental health challenges. Finally, trauma may be a key factor influencing both mental health and substance use among adolescents. Future research may seek to explore the role of adverse childhood experiences (29), community-level trauma, and historical factors in relation to adolescent mental health and substance use initiation.

Finally, although not a limitation in achieving our scientific aims, we caution against overgeneralization of these findings, and we encourage future prevention research with AI communities to emphasize a strengths-based perspective given underutilized cultural assets (10, 12). The tribes represented in this study are a small percentage of the heterogeneity of adolescents indigenous to North America. While reported findings provide critical insight into a gap in etiologic evidence on mental health and substance use initiation among rural youth, our sample composition is unique to the Cherokee Nation Reservation and bordering areas. Approximately half of the participants identified as AI only or AI/White only, and 36%–39% as White only. It is important to consider that a significant majority of Native adolescents in the U.S. are not reservation-based, maintain different cultural identities, and carry strengths that are distinct from those of our participants (59, 60). Further, while this study examines the challenges related to mental health and substance use initiation, the findings offer valuable insights that can inform culturally relevant interventions for rural and AI youth. Though not the focus of this study, the cultural strengths of AI communities—such as community, tradition, and resilience—are important considerations for future prevention efforts with these populations (10, 12).

Directions for research and intervention pertain to the shifting norms around cannabis and vaping. Our results show that mental health problems heighten the odds of initiating certain substances. Mental health may therefore be a critical intervention target in efforts to prevent initiation of common substances, including those of increasing popularity among adolescents. Cannabis is now the most common first substance of use among AI adolescents of a Northern Plains tribe, even before alcohol (24). Vaping has significantly increased among adolescents of the present study's Tribal reservation (19% to 29%) between 2017 and 2019 (61). Given the attention to culturally relevant interventions for AI/AN and other adolescents living in rural areas (10, 12, 42), a sharper understanding of timing, proximal risk factors, and motivations for initiation (e.g., self-medication) might be helpful to inform intervention development.

In conclusion, this study's implications for prevention among AI and other adolescents in rural communities underscore the importance of early intervention in mental health issues to mitigate substance use initiation. This research significantly contributes to the evidence base by illuminating initiation risks within a population often marginalized in academic literature. Study insights provide a foundation for more targeted efforts to prevent mental health and subsequent substance use challenges faced by AI and other adolescents living in rural communities.

## Data Availability

The datasets presented in this article are not readily available because we are abiding with relevant NIH policies, laws and regulations, while simultaneously protecting the identity and confidentiality of the study sample and the partner Tribal Nation, a small socially identifiable population. Federally recognized tribes enjoy special protections as domestic dependent sovereignties. As such, any entity other than this Tribal Nation wishing to access, view or use the data from this study will need to request explicit permission from the Tribal Nation and must agree to abide by the procedures and requirements of the Tribal Nation's IRB review process. Requests to access the datasets should be directed to irb@cherokee.org.
